# Supplementary material for: Optimal minimal residual disease threshold in pediatric acute myeloid leukemia: A retrospective cohort study based on the TARGET database
Source: PLoS Med. 2026 May 8;23(5):e1005088. doi: 10.1371/journal.pmed.1005088 (PMC13155632; doi:10.1371/journal.pmed.1005088)
Supplement: S1 Code — (ZIP) [file pmed.1005088.s002.zip › S2 code/PROJ8_16_tbl2/PROJ8_16_tbl2.htm]

## Kaplan Meier Survival Curve

Outcome: Vital Status
Time: OS(years)
Landmark analysis for time segment: OS(years) <= 5
Log rank test: implements the G-rho family of Harrington and Fleming (1982), with weights on each death of S(t)^rho, where S is the Kaplan-Meier estimate of survival. With rho = 0 this is the log-rank or Mantel-Haenszel test.

|  |  |  |  |
| --- | --- | --- | --- |
|  | N | Observed | Expected |
| factor(X25.CS)=0 | 467 | 54 | 57.3217 |
| factor(X25.CS)=1 | 16 | 5 | 1.6783 |

Chisq=
6.7764
on
1
degree of freedom, p=
0.0092
Survival table

|  |  |  |  |  |  |  |  |
| --- | --- | --- | --- | --- | --- | --- | --- |
|  | OS(years) | N.Risk | N.Event | N.Censor | Survival | 95%CI Low | 95%CI Upp |
| factor(X25.CS)=0 | 0.23 | 467 | 2 | 0 | 0.9957 | 0.9898 | 1.0000 |
| factor(X25.CS)=0 | 0.26 | 465 | 1 | 0 | 0.9936 | 0.9864 | 1.0000 |
| factor(X25.CS)=0 | 0.33 | 464 | 1 | 0 | 0.9914 | 0.9831 | 0.9998 |
| factor(X25.CS)=0 | 0.41 | 463 | 2 | 0 | 0.9872 | 0.9770 | 0.9974 |
| factor(X25.CS)=0 | 0.42 | 461 | 1 | 0 | 0.9850 | 0.9741 | 0.9961 |
| factor(X25.CS)=0 | 0.44 | 460 | 1 | 0 | 0.9829 | 0.9712 | 0.9947 |
| factor(X25.CS)=0 | 0.5 | 459 | 1 | 0 | 0.9807 | 0.9683 | 0.9933 |
| factor(X25.CS)=0 | 0.52 | 458 | 1 | 0 | 0.9786 | 0.9655 | 0.9918 |
| factor(X25.CS)=0 | 0.57 | 457 | 2 | 0 | 0.9743 | 0.9601 | 0.9888 |
| factor(X25.CS)=0 | 0.58 | 455 | 1 | 0 | 0.9722 | 0.9574 | 0.9872 |
| factor(X25.CS)=0 | 0.68 | 454 | 3 | 0 | 0.9657 | 0.9494 | 0.9824 |
| factor(X25.CS)=0 | 0.79 | 451 | 1 | 0 | 0.9636 | 0.9468 | 0.9807 |
| factor(X25.CS)=0 | 0.81 | 450 | 1 | 0 | 0.9615 | 0.9442 | 0.9791 |
| factor(X25.CS)=0 | 0.86 | 449 | 1 | 0 | 0.9593 | 0.9416 | 0.9774 |
| factor(X25.CS)=0 | 0.97 | 448 | 2 | 0 | 0.9550 | 0.9364 | 0.9740 |
| factor(X25.CS)=0 | 0.99 | 446 | 0 | 1 | 0.9550 | 0.9364 | 0.9740 |
| factor(X25.CS)=0 | 1.06 | 445 | 0 | 1 | 0.9550 | 0.9364 | 0.9740 |
| factor(X25.CS)=0 | 1.07 | 444 | 1 | 0 | 0.9529 | 0.9339 | 0.9723 |
| factor(X25.CS)=0 | 1.1 | 443 | 1 | 0 | 0.9507 | 0.9313 | 0.9706 |
| factor(X25.CS)=0 | 1.12 | 442 | 0 | 1 | 0.9507 | 0.9313 | 0.9706 |
| factor(X25.CS)=0 | 1.13 | 441 | 1 | 0 | 0.9486 | 0.9287 | 0.9688 |
| factor(X25.CS)=0 | 1.14 | 440 | 0 | 1 | 0.9486 | 0.9287 | 0.9688 |
| factor(X25.CS)=0 | 1.17 | 439 | 0 | 1 | 0.9486 | 0.9287 | 0.9688 |
| factor(X25.CS)=0 | 1.19 | 438 | 1 | 0 | 0.9464 | 0.9262 | 0.9671 |
| factor(X25.CS)=0 | 1.21 | 437 | 1 | 0 | 0.9442 | 0.9236 | 0.9653 |
| factor(X25.CS)=0 | 1.28 | 436 | 0 | 1 | 0.9442 | 0.9236 | 0.9653 |
| factor(X25.CS)=0 | 1.34 | 435 | 0 | 2 | 0.9442 | 0.9236 | 0.9653 |
| factor(X25.CS)=0 | 1.42 | 433 | 1 | 0 | 0.9421 | 0.9211 | 0.9635 |
| factor(X25.CS)=0 | 1.45 | 432 | 2 | 0 | 0.9377 | 0.9160 | 0.9599 |
| factor(X25.CS)=0 | 1.52 | 430 | 1 | 0 | 0.9355 | 0.9135 | 0.9581 |
| factor(X25.CS)=0 | 1.55 | 429 | 1 | 0 | 0.9333 | 0.9109 | 0.9563 |
| factor(X25.CS)=0 | 1.56 | 428 | 1 | 0 | 0.9312 | 0.9084 | 0.9545 |
| factor(X25.CS)=0 | 1.6 | 427 | 1 | 0 | 0.9290 | 0.9059 | 0.9526 |
| factor(X25.CS)=0 | 1.71 | 426 | 0 | 1 | 0.9290 | 0.9059 | 0.9526 |
| factor(X25.CS)=0 | 1.72 | 425 | 1 | 0 | 0.9268 | 0.9034 | 0.9508 |
| factor(X25.CS)=0 | 1.76 | 424 | 1 | 0 | 0.9246 | 0.9009 | 0.9489 |
| factor(X25.CS)=0 | 1.78 | 423 | 1 | 0 | 0.9224 | 0.8984 | 0.9471 |
| factor(X25.CS)=0 | 1.87 | 422 | 0 | 1 | 0.9224 | 0.8984 | 0.9471 |
| factor(X25.CS)=0 | 1.91 | 421 | 2 | 0 | 0.9180 | 0.8934 | 0.9434 |
| factor(X25.CS)=0 | 1.92 | 419 | 0 | 1 | 0.9180 | 0.8934 | 0.9434 |
| factor(X25.CS)=0 | 1.94 | 418 | 0 | 2 | 0.9180 | 0.8934 | 0.9434 |
| factor(X25.CS)=0 | 1.96 | 416 | 0 | 1 | 0.9180 | 0.8934 | 0.9434 |
| factor(X25.CS)=0 | 1.97 | 415 | 0 | 1 | 0.9180 | 0.8934 | 0.9434 |
| factor(X25.CS)=0 | 1.98 | 414 | 2 | 1 | 0.9136 | 0.8884 | 0.9396 |
| factor(X25.CS)=0 | 2.1 | 411 | 0 | 1 | 0.9136 | 0.8884 | 0.9396 |
| factor(X25.CS)=0 | 2.11 | 410 | 0 | 1 | 0.9136 | 0.8884 | 0.9396 |
| factor(X25.CS)=0 | 2.21 | 409 | 1 | 0 | 0.9114 | 0.8858 | 0.9376 |
| factor(X25.CS)=0 | 2.22 | 408 | 0 | 1 | 0.9114 | 0.8858 | 0.9376 |
| factor(X25.CS)=0 | 2.27 | 407 | 0 | 3 | 0.9114 | 0.8858 | 0.9376 |
| factor(X25.CS)=0 | 2.31 | 404 | 1 | 0 | 0.9091 | 0.8833 | 0.9357 |
| factor(X25.CS)=0 | 2.32 | 403 | 0 | 2 | 0.9091 | 0.8833 | 0.9357 |
| factor(X25.CS)=0 | 2.34 | 401 | 0 | 1 | 0.9091 | 0.8833 | 0.9357 |
| factor(X25.CS)=0 | 2.37 | 400 | 0 | 1 | 0.9091 | 0.8833 | 0.9357 |
| factor(X25.CS)=0 | 2.39 | 399 | 0 | 2 | 0.9091 | 0.8833 | 0.9357 |
| factor(X25.CS)=0 | 2.4 | 397 | 0 | 1 | 0.9091 | 0.8833 | 0.9357 |
| factor(X25.CS)=0 | 2.41 | 396 | 0 | 2 | 0.9091 | 0.8833 | 0.9357 |
| factor(X25.CS)=0 | 2.44 | 394 | 0 | 2 | 0.9091 | 0.8833 | 0.9357 |
| factor(X25.CS)=0 | 2.47 | 392 | 0 | 1 | 0.9091 | 0.8833 | 0.9357 |
| factor(X25.CS)=0 | 2.49 | 391 | 0 | 1 | 0.9091 | 0.8833 | 0.9357 |
| factor(X25.CS)=0 | 2.5 | 390 | 0 | 2 | 0.9091 | 0.8833 | 0.9357 |
| factor(X25.CS)=0 | 2.53 | 388 | 1 | 2 | 0.9068 | 0.8806 | 0.9337 |
| factor(X25.CS)=0 | 2.55 | 385 | 0 | 2 | 0.9068 | 0.8806 | 0.9337 |
| factor(X25.CS)=0 | 2.64 | 383 | 0 | 1 | 0.9068 | 0.8806 | 0.9337 |
| factor(X25.CS)=0 | 2.65 | 382 | 0 | 1 | 0.9068 | 0.8806 | 0.9337 |
| factor(X25.CS)=0 | 2.68 | 381 | 0 | 1 | 0.9068 | 0.8806 | 0.9337 |
| factor(X25.CS)=0 | 2.7 | 380 | 0 | 1 | 0.9068 | 0.8806 | 0.9337 |
| factor(X25.CS)=0 | 2.75 | 379 | 0 | 1 | 0.9068 | 0.8806 | 0.9337 |
| factor(X25.CS)=0 | 2.79 | 378 | 0 | 1 | 0.9068 | 0.8806 | 0.9337 |
| factor(X25.CS)=0 | 2.83 | 377 | 0 | 1 | 0.9068 | 0.8806 | 0.9337 |
| factor(X25.CS)=0 | 2.86 | 376 | 1 | 0 | 0.9044 | 0.8779 | 0.9317 |
| factor(X25.CS)=0 | 2.93 | 375 | 0 | 1 | 0.9044 | 0.8779 | 0.9317 |
| factor(X25.CS)=0 | 2.95 | 374 | 0 | 1 | 0.9044 | 0.8779 | 0.9317 |
| factor(X25.CS)=0 | 2.96 | 373 | 1 | 0 | 0.9019 | 0.8751 | 0.9296 |
| factor(X25.CS)=0 | 3.01 | 372 | 1 | 0 | 0.8995 | 0.8723 | 0.9275 |
| factor(X25.CS)=0 | 3.02 | 371 | 0 | 1 | 0.8995 | 0.8723 | 0.9275 |
| factor(X25.CS)=0 | 3.03 | 370 | 0 | 1 | 0.8995 | 0.8723 | 0.9275 |
| factor(X25.CS)=0 | 3.05 | 369 | 0 | 1 | 0.8995 | 0.8723 | 0.9275 |
| factor(X25.CS)=0 | 3.09 | 368 | 1 | 1 | 0.8971 | 0.8696 | 0.9254 |
| factor(X25.CS)=0 | 3.1 | 366 | 1 | 0 | 0.8946 | 0.8668 | 0.9233 |
| factor(X25.CS)=0 | 3.11 | 365 | 0 | 1 | 0.8946 | 0.8668 | 0.9233 |
| factor(X25.CS)=0 | 3.14 | 364 | 1 | 1 | 0.8922 | 0.8640 | 0.9212 |
| factor(X25.CS)=0 | 3.15 | 362 | 0 | 1 | 0.8922 | 0.8640 | 0.9212 |
| factor(X25.CS)=0 | 3.16 | 361 | 0 | 1 | 0.8922 | 0.8640 | 0.9212 |
| factor(X25.CS)=0 | 3.17 | 360 | 0 | 1 | 0.8922 | 0.8640 | 0.9212 |
| factor(X25.CS)=0 | 3.19 | 359 | 0 | 1 | 0.8922 | 0.8640 | 0.9212 |
| factor(X25.CS)=0 | 3.2 | 358 | 0 | 1 | 0.8922 | 0.8640 | 0.9212 |
| factor(X25.CS)=0 | 3.21 | 357 | 0 | 1 | 0.8922 | 0.8640 | 0.9212 |
| factor(X25.CS)=0 | 3.22 | 356 | 0 | 1 | 0.8922 | 0.8640 | 0.9212 |
| factor(X25.CS)=0 | 3.23 | 355 | 0 | 2 | 0.8922 | 0.8640 | 0.9212 |
| factor(X25.CS)=0 | 3.24 | 353 | 0 | 1 | 0.8922 | 0.8640 | 0.9212 |
| factor(X25.CS)=0 | 3.25 | 352 | 0 | 1 | 0.8922 | 0.8640 | 0.9212 |
| factor(X25.CS)=0 | 3.26 | 351 | 0 | 1 | 0.8922 | 0.8640 | 0.9212 |
| factor(X25.CS)=0 | 3.28 | 350 | 0 | 1 | 0.8922 | 0.8640 | 0.9212 |
| factor(X25.CS)=0 | 3.3 | 349 | 0 | 1 | 0.8922 | 0.8640 | 0.9212 |
| factor(X25.CS)=0 | 3.32 | 348 | 0 | 1 | 0.8922 | 0.8640 | 0.9212 |
| factor(X25.CS)=0 | 3.33 | 347 | 0 | 2 | 0.8922 | 0.8640 | 0.9212 |
| factor(X25.CS)=0 | 3.34 | 345 | 0 | 2 | 0.8922 | 0.8640 | 0.9212 |
| factor(X25.CS)=0 | 3.35 | 343 | 0 | 2 | 0.8922 | 0.8640 | 0.9212 |
| factor(X25.CS)=0 | 3.36 | 341 | 0 | 1 | 0.8922 | 0.8640 | 0.9212 |
| factor(X25.CS)=0 | 3.38 | 340 | 0 | 2 | 0.8922 | 0.8640 | 0.9212 |
| factor(X25.CS)=0 | 3.39 | 338 | 0 | 2 | 0.8922 | 0.8640 | 0.9212 |
| factor(X25.CS)=0 | 3.4 | 336 | 0 | 1 | 0.8922 | 0.8640 | 0.9212 |
| factor(X25.CS)=0 | 3.41 | 335 | 0 | 1 | 0.8922 | 0.8640 | 0.9212 |
| factor(X25.CS)=0 | 3.43 | 334 | 0 | 1 | 0.8922 | 0.8640 | 0.9212 |
| factor(X25.CS)=0 | 3.44 | 333 | 0 | 1 | 0.8922 | 0.8640 | 0.9212 |
| factor(X25.CS)=0 | 3.45 | 332 | 0 | 2 | 0.8922 | 0.8640 | 0.9212 |
| factor(X25.CS)=0 | 3.5 | 330 | 0 | 2 | 0.8922 | 0.8640 | 0.9212 |
| factor(X25.CS)=0 | 3.52 | 328 | 0 | 1 | 0.8922 | 0.8640 | 0.9212 |
| factor(X25.CS)=0 | 3.54 | 327 | 0 | 1 | 0.8922 | 0.8640 | 0.9212 |
| factor(X25.CS)=0 | 3.55 | 326 | 0 | 1 | 0.8922 | 0.8640 | 0.9212 |
| factor(X25.CS)=0 | 3.56 | 325 | 0 | 3 | 0.8922 | 0.8640 | 0.9212 |
| factor(X25.CS)=0 | 3.57 | 322 | 0 | 1 | 0.8922 | 0.8640 | 0.9212 |
| factor(X25.CS)=0 | 3.58 | 321 | 0 | 2 | 0.8922 | 0.8640 | 0.9212 |
| factor(X25.CS)=0 | 3.61 | 319 | 0 | 1 | 0.8922 | 0.8640 | 0.9212 |
| factor(X25.CS)=0 | 3.64 | 318 | 1 | 0 | 0.8894 | 0.8608 | 0.9188 |
| factor(X25.CS)=0 | 3.65 | 317 | 1 | 2 | 0.8865 | 0.8576 | 0.9165 |
| factor(X25.CS)=0 | 3.66 | 314 | 0 | 1 | 0.8865 | 0.8576 | 0.9165 |
| factor(X25.CS)=0 | 3.72 | 313 | 0 | 1 | 0.8865 | 0.8576 | 0.9165 |
| factor(X25.CS)=0 | 3.79 | 312 | 0 | 1 | 0.8865 | 0.8576 | 0.9165 |
| factor(X25.CS)=0 | 3.8 | 311 | 1 | 0 | 0.8837 | 0.8543 | 0.9141 |
| factor(X25.CS)=0 | 3.81 | 310 | 0 | 1 | 0.8837 | 0.8543 | 0.9141 |
| factor(X25.CS)=0 | 3.83 | 309 | 0 | 1 | 0.8837 | 0.8543 | 0.9141 |
| factor(X25.CS)=0 | 3.9 | 308 | 0 | 1 | 0.8837 | 0.8543 | 0.9141 |
| factor(X25.CS)=0 | 3.95 | 307 | 0 | 1 | 0.8837 | 0.8543 | 0.9141 |
| factor(X25.CS)=0 | 4.01 | 306 | 0 | 1 | 0.8837 | 0.8543 | 0.9141 |
| factor(X25.CS)=0 | 4.02 | 305 | 0 | 1 | 0.8837 | 0.8543 | 0.9141 |
| factor(X25.CS)=0 | 4.05 | 304 | 0 | 1 | 0.8837 | 0.8543 | 0.9141 |
| factor(X25.CS)=0 | 4.06 | 303 | 0 | 2 | 0.8837 | 0.8543 | 0.9141 |
| factor(X25.CS)=0 | 4.07 | 301 | 0 | 1 | 0.8837 | 0.8543 | 0.9141 |
| factor(X25.CS)=0 | 4.08 | 300 | 0 | 1 | 0.8837 | 0.8543 | 0.9141 |
| factor(X25.CS)=0 | 4.09 | 299 | 0 | 1 | 0.8837 | 0.8543 | 0.9141 |
| factor(X25.CS)=0 | 4.12 | 298 | 0 | 1 | 0.8837 | 0.8543 | 0.9141 |
| factor(X25.CS)=0 | 4.13 | 297 | 0 | 2 | 0.8837 | 0.8543 | 0.9141 |
| factor(X25.CS)=0 | 4.15 | 295 | 0 | 2 | 0.8837 | 0.8543 | 0.9141 |
| factor(X25.CS)=0 | 4.16 | 293 | 0 | 2 | 0.8837 | 0.8543 | 0.9141 |
| factor(X25.CS)=0 | 4.26 | 291 | 0 | 1 | 0.8837 | 0.8543 | 0.9141 |
| factor(X25.CS)=0 | 4.27 | 290 | 0 | 1 | 0.8837 | 0.8543 | 0.9141 |
| factor(X25.CS)=0 | 4.31 | 289 | 0 | 1 | 0.8837 | 0.8543 | 0.9141 |
| factor(X25.CS)=0 | 4.32 | 288 | 0 | 1 | 0.8837 | 0.8543 | 0.9141 |
| factor(X25.CS)=0 | 4.33 | 287 | 0 | 1 | 0.8837 | 0.8543 | 0.9141 |
| factor(X25.CS)=0 | 4.35 | 286 | 0 | 4 | 0.8837 | 0.8543 | 0.9141 |
| factor(X25.CS)=0 | 4.36 | 282 | 0 | 4 | 0.8837 | 0.8543 | 0.9141 |
| factor(X25.CS)=0 | 4.37 | 278 | 0 | 1 | 0.8837 | 0.8543 | 0.9141 |
| factor(X25.CS)=0 | 4.38 | 277 | 0 | 1 | 0.8837 | 0.8543 | 0.9141 |
| factor(X25.CS)=0 | 4.4 | 276 | 0 | 1 | 0.8837 | 0.8543 | 0.9141 |
| factor(X25.CS)=0 | 4.41 | 275 | 0 | 1 | 0.8837 | 0.8543 | 0.9141 |
| factor(X25.CS)=0 | 4.42 | 274 | 0 | 1 | 0.8837 | 0.8543 | 0.9141 |
| factor(X25.CS)=0 | 4.43 | 273 | 0 | 1 | 0.8837 | 0.8543 | 0.9141 |
| factor(X25.CS)=0 | 4.44 | 272 | 0 | 1 | 0.8837 | 0.8543 | 0.9141 |
| factor(X25.CS)=0 | 4.47 | 271 | 0 | 1 | 0.8837 | 0.8543 | 0.9141 |
| factor(X25.CS)=0 | 4.48 | 270 | 0 | 1 | 0.8837 | 0.8543 | 0.9141 |
| factor(X25.CS)=0 | 4.53 | 269 | 0 | 1 | 0.8837 | 0.8543 | 0.9141 |
| factor(X25.CS)=0 | 4.54 | 268 | 0 | 2 | 0.8837 | 0.8543 | 0.9141 |
| factor(X25.CS)=0 | 4.55 | 266 | 0 | 1 | 0.8837 | 0.8543 | 0.9141 |
| factor(X25.CS)=0 | 4.59 | 265 | 0 | 1 | 0.8837 | 0.8543 | 0.9141 |
| factor(X25.CS)=0 | 4.6 | 264 | 0 | 2 | 0.8837 | 0.8543 | 0.9141 |
| factor(X25.CS)=0 | 4.61 | 262 | 0 | 1 | 0.8837 | 0.8543 | 0.9141 |
| factor(X25.CS)=0 | 4.64 | 261 | 0 | 1 | 0.8837 | 0.8543 | 0.9141 |
| factor(X25.CS)=0 | 4.68 | 260 | 0 | 1 | 0.8837 | 0.8543 | 0.9141 |
| factor(X25.CS)=0 | 4.69 | 259 | 0 | 1 | 0.8837 | 0.8543 | 0.9141 |
| factor(X25.CS)=0 | 4.72 | 258 | 0 | 1 | 0.8837 | 0.8543 | 0.9141 |
| factor(X25.CS)=0 | 4.73 | 257 | 0 | 1 | 0.8837 | 0.8543 | 0.9141 |
| factor(X25.CS)=0 | 4.74 | 256 | 1 | 0 | 0.8802 | 0.8503 | 0.9113 |
| factor(X25.CS)=0 | 4.75 | 255 | 0 | 1 | 0.8802 | 0.8503 | 0.9113 |
| factor(X25.CS)=0 | 4.81 | 254 | 0 | 2 | 0.8802 | 0.8503 | 0.9113 |
| factor(X25.CS)=0 | 4.82 | 252 | 0 | 1 | 0.8802 | 0.8503 | 0.9113 |
| factor(X25.CS)=0 | 4.84 | 251 | 1 | 0 | 0.8767 | 0.8461 | 0.9085 |
| factor(X25.CS)=0 | 4.85 | 250 | 0 | 1 | 0.8767 | 0.8461 | 0.9085 |
| factor(X25.CS)=0 | 4.88 | 249 | 0 | 1 | 0.8767 | 0.8461 | 0.9085 |
| factor(X25.CS)=0 | 4.89 | 248 | 0 | 1 | 0.8767 | 0.8461 | 0.9085 |
| factor(X25.CS)=0 | 4.91 | 247 | 0 | 1 | 0.8767 | 0.8461 | 0.9085 |
| factor(X25.CS)=0 | 4.94 | 246 | 0 | 2 | 0.8767 | 0.8461 | 0.9085 |
| factor(X25.CS)=0 | 4.96 | 244 | 0 | 1 | 0.8767 | 0.8461 | 0.9085 |
| factor(X25.CS)=0 | 4.97 | 243 | 0 | 1 | 0.8767 | 0.8461 | 0.9085 |
| factor(X25.CS)=0 | 5 | 242 | 0 | 242 | 0.8767 | 0.8461 | 0.9085 |
| factor(X25.CS)=1 | 0.47 | 16 | 2 | 0 | 0.8750 | 0.7271 | 1.0000 |
| factor(X25.CS)=1 | 0.86 | 14 | 0 | 1 | 0.8750 | 0.7271 | 1.0000 |
| factor(X25.CS)=1 | 1.03 | 13 | 1 | 0 | 0.8077 | 0.6336 | 1.0000 |
| factor(X25.CS)=1 | 1.19 | 12 | 1 | 0 | 0.7404 | 0.5503 | 0.9961 |
| factor(X25.CS)=1 | 1.94 | 11 | 1 | 0 | 0.6731 | 0.4740 | 0.9558 |
| factor(X25.CS)=1 | 5 | 10 | 0 | 10 | 0.6731 | 0.4740 | 0.9558 |

|  |  |  |  |  |  |  |  |  |  |
| --- | --- | --- | --- | --- | --- | --- | --- | --- | --- |
| X25.CS | records | n.max | n.start | events | \*rmean | \*se(rmean) | median | 0.95LCL | 0.95UCL |
| factor(X25.CS)=0 | 467 | 467 | 467 | 54 | 4.598 | 0.054 | NA | NA | NA |
| factor(X25.CS)=1 | 16 | 16 | 16 | 5 | 3.704 | 0.482 | NA | 1.94 | NA |

Landmark analysis for time segment: OS(years) > 5
Log rank test: implements the G-rho family of Harrington and Fleming (1982), with weights on each death of S(t)^rho, where S is the Kaplan-Meier estimate of survival. With rho = 0 this is the log-rank or Mantel-Haenszel test.

|  |  |  |  |
| --- | --- | --- | --- |
|  | N | Observed | Expected |
| factor(X25.CS)=0 | 241 | 2 | 1.8317 |
| factor(X25.CS)=1 | 10 | 0 | 0.1683 |

Chisq=
0.1878
on
1
degree of freedom, p=
0.6648
Survival table

|  |  |  |  |  |  |  |  |
| --- | --- | --- | --- | --- | --- | --- | --- |
|  | OS(years) | N.Risk | N.Event | N.Censor | Survival | 95%CI Low | 95%CI Upp |
| factor(X25.CS)=0 | 5.02 | 241 | 0 | 1 | 1.0000 | 1.0000 | 1.0000 |
| factor(X25.CS)=0 | 5.03 | 240 | 0 | 1 | 1.0000 | 1.0000 | 1.0000 |
| factor(X25.CS)=0 | 5.04 | 239 | 0 | 1 | 1.0000 | 1.0000 | 1.0000 |
| factor(X25.CS)=0 | 5.05 | 238 | 0 | 1 | 1.0000 | 1.0000 | 1.0000 |
| factor(X25.CS)=0 | 5.08 | 237 | 0 | 2 | 1.0000 | 1.0000 | 1.0000 |
| factor(X25.CS)=0 | 5.09 | 235 | 0 | 1 | 1.0000 | 1.0000 | 1.0000 |
| factor(X25.CS)=0 | 5.1 | 234 | 0 | 1 | 1.0000 | 1.0000 | 1.0000 |
| factor(X25.CS)=0 | 5.12 | 233 | 0 | 2 | 1.0000 | 1.0000 | 1.0000 |
| factor(X25.CS)=0 | 5.15 | 231 | 0 | 3 | 1.0000 | 1.0000 | 1.0000 |
| factor(X25.CS)=0 | 5.16 | 228 | 0 | 1 | 1.0000 | 1.0000 | 1.0000 |
| factor(X25.CS)=0 | 5.17 | 227 | 0 | 1 | 1.0000 | 1.0000 | 1.0000 |
| factor(X25.CS)=0 | 5.18 | 226 | 0 | 3 | 1.0000 | 1.0000 | 1.0000 |
| factor(X25.CS)=0 | 5.22 | 223 | 0 | 2 | 1.0000 | 1.0000 | 1.0000 |
| factor(X25.CS)=0 | 5.25 | 221 | 0 | 2 | 1.0000 | 1.0000 | 1.0000 |
| factor(X25.CS)=0 | 5.26 | 219 | 0 | 2 | 1.0000 | 1.0000 | 1.0000 |
| factor(X25.CS)=0 | 5.29 | 217 | 0 | 3 | 1.0000 | 1.0000 | 1.0000 |
| factor(X25.CS)=0 | 5.31 | 214 | 0 | 1 | 1.0000 | 1.0000 | 1.0000 |
| factor(X25.CS)=0 | 5.32 | 213 | 0 | 1 | 1.0000 | 1.0000 | 1.0000 |
| factor(X25.CS)=0 | 5.33 | 212 | 0 | 1 | 1.0000 | 1.0000 | 1.0000 |
| factor(X25.CS)=0 | 5.34 | 211 | 0 | 2 | 1.0000 | 1.0000 | 1.0000 |
| factor(X25.CS)=0 | 5.35 | 209 | 0 | 3 | 1.0000 | 1.0000 | 1.0000 |
| factor(X25.CS)=0 | 5.36 | 206 | 0 | 1 | 1.0000 | 1.0000 | 1.0000 |
| factor(X25.CS)=0 | 5.38 | 205 | 0 | 1 | 1.0000 | 1.0000 | 1.0000 |
| factor(X25.CS)=0 | 5.39 | 204 | 0 | 2 | 1.0000 | 1.0000 | 1.0000 |
| factor(X25.CS)=0 | 5.4 | 202 | 0 | 1 | 1.0000 | 1.0000 | 1.0000 |
| factor(X25.CS)=0 | 5.41 | 201 | 0 | 2 | 1.0000 | 1.0000 | 1.0000 |
| factor(X25.CS)=0 | 5.42 | 199 | 1 | 4 | 0.9950 | 0.9852 | 1.0000 |
| factor(X25.CS)=0 | 5.43 | 194 | 0 | 1 | 0.9950 | 0.9852 | 1.0000 |
| factor(X25.CS)=0 | 5.44 | 193 | 0 | 3 | 0.9950 | 0.9852 | 1.0000 |
| factor(X25.CS)=0 | 5.45 | 190 | 0 | 1 | 0.9950 | 0.9852 | 1.0000 |
| factor(X25.CS)=0 | 5.46 | 189 | 0 | 1 | 0.9950 | 0.9852 | 1.0000 |
| factor(X25.CS)=0 | 5.48 | 188 | 0 | 3 | 0.9950 | 0.9852 | 1.0000 |
| factor(X25.CS)=0 | 5.49 | 185 | 0 | 1 | 0.9950 | 0.9852 | 1.0000 |
| factor(X25.CS)=0 | 5.5 | 184 | 0 | 1 | 0.9950 | 0.9852 | 1.0000 |
| factor(X25.CS)=0 | 5.51 | 183 | 0 | 2 | 0.9950 | 0.9852 | 1.0000 |
| factor(X25.CS)=0 | 5.52 | 181 | 0 | 1 | 0.9950 | 0.9852 | 1.0000 |
| factor(X25.CS)=0 | 5.54 | 180 | 0 | 2 | 0.9950 | 0.9852 | 1.0000 |
| factor(X25.CS)=0 | 5.55 | 178 | 0 | 1 | 0.9950 | 0.9852 | 1.0000 |
| factor(X25.CS)=0 | 5.56 | 177 | 0 | 2 | 0.9950 | 0.9852 | 1.0000 |
| factor(X25.CS)=0 | 5.57 | 175 | 0 | 1 | 0.9950 | 0.9852 | 1.0000 |
| factor(X25.CS)=0 | 5.58 | 174 | 0 | 2 | 0.9950 | 0.9852 | 1.0000 |
| factor(X25.CS)=0 | 5.63 | 172 | 0 | 1 | 0.9950 | 0.9852 | 1.0000 |
| factor(X25.CS)=0 | 5.65 | 171 | 0 | 1 | 0.9950 | 0.9852 | 1.0000 |
| factor(X25.CS)=0 | 5.66 | 170 | 0 | 1 | 0.9950 | 0.9852 | 1.0000 |
| factor(X25.CS)=0 | 5.67 | 169 | 0 | 2 | 0.9950 | 0.9852 | 1.0000 |
| factor(X25.CS)=0 | 5.69 | 167 | 0 | 1 | 0.9950 | 0.9852 | 1.0000 |
| factor(X25.CS)=0 | 5.7 | 166 | 0 | 1 | 0.9950 | 0.9852 | 1.0000 |
| factor(X25.CS)=0 | 5.71 | 165 | 0 | 2 | 0.9950 | 0.9852 | 1.0000 |
| factor(X25.CS)=0 | 5.72 | 163 | 0 | 1 | 0.9950 | 0.9852 | 1.0000 |
| factor(X25.CS)=0 | 5.73 | 162 | 0 | 2 | 0.9950 | 0.9852 | 1.0000 |
| factor(X25.CS)=0 | 5.75 | 160 | 0 | 1 | 0.9950 | 0.9852 | 1.0000 |
| factor(X25.CS)=0 | 5.77 | 159 | 0 | 1 | 0.9950 | 0.9852 | 1.0000 |
| factor(X25.CS)=0 | 5.78 | 158 | 0 | 2 | 0.9950 | 0.9852 | 1.0000 |
| factor(X25.CS)=0 | 5.84 | 156 | 0 | 1 | 0.9950 | 0.9852 | 1.0000 |
| factor(X25.CS)=0 | 5.85 | 155 | 0 | 1 | 0.9950 | 0.9852 | 1.0000 |
| factor(X25.CS)=0 | 5.88 | 154 | 0 | 1 | 0.9950 | 0.9852 | 1.0000 |
| factor(X25.CS)=0 | 5.89 | 153 | 0 | 2 | 0.9950 | 0.9852 | 1.0000 |
| factor(X25.CS)=0 | 5.92 | 151 | 0 | 1 | 0.9950 | 0.9852 | 1.0000 |
| factor(X25.CS)=0 | 5.93 | 150 | 0 | 1 | 0.9950 | 0.9852 | 1.0000 |
| factor(X25.CS)=0 | 5.94 | 149 | 0 | 1 | 0.9950 | 0.9852 | 1.0000 |
| factor(X25.CS)=0 | 5.95 | 148 | 0 | 3 | 0.9950 | 0.9852 | 1.0000 |
| factor(X25.CS)=0 | 5.96 | 145 | 0 | 1 | 0.9950 | 0.9852 | 1.0000 |
| factor(X25.CS)=0 | 5.98 | 144 | 0 | 1 | 0.9950 | 0.9852 | 1.0000 |
| factor(X25.CS)=0 | 6 | 143 | 0 | 4 | 0.9950 | 0.9852 | 1.0000 |
| factor(X25.CS)=0 | 6.01 | 139 | 0 | 2 | 0.9950 | 0.9852 | 1.0000 |
| factor(X25.CS)=0 | 6.02 | 137 | 0 | 2 | 0.9950 | 0.9852 | 1.0000 |
| factor(X25.CS)=0 | 6.04 | 135 | 0 | 1 | 0.9950 | 0.9852 | 1.0000 |
| factor(X25.CS)=0 | 6.05 | 134 | 0 | 1 | 0.9950 | 0.9852 | 1.0000 |
| factor(X25.CS)=0 | 6.07 | 133 | 0 | 1 | 0.9950 | 0.9852 | 1.0000 |
| factor(X25.CS)=0 | 6.08 | 132 | 0 | 3 | 0.9950 | 0.9852 | 1.0000 |
| factor(X25.CS)=0 | 6.12 | 129 | 0 | 2 | 0.9950 | 0.9852 | 1.0000 |
| factor(X25.CS)=0 | 6.14 | 127 | 0 | 2 | 0.9950 | 0.9852 | 1.0000 |
| factor(X25.CS)=0 | 6.15 | 125 | 0 | 3 | 0.9950 | 0.9852 | 1.0000 |
| factor(X25.CS)=0 | 6.16 | 122 | 0 | 2 | 0.9950 | 0.9852 | 1.0000 |
| factor(X25.CS)=0 | 6.18 | 120 | 0 | 2 | 0.9950 | 0.9852 | 1.0000 |
| factor(X25.CS)=0 | 6.22 | 118 | 0 | 1 | 0.9950 | 0.9852 | 1.0000 |
| factor(X25.CS)=0 | 6.25 | 117 | 0 | 2 | 0.9950 | 0.9852 | 1.0000 |
| factor(X25.CS)=0 | 6.27 | 115 | 0 | 1 | 0.9950 | 0.9852 | 1.0000 |
| factor(X25.CS)=0 | 6.28 | 114 | 0 | 1 | 0.9950 | 0.9852 | 1.0000 |
| factor(X25.CS)=0 | 6.29 | 113 | 0 | 2 | 0.9950 | 0.9852 | 1.0000 |
| factor(X25.CS)=0 | 6.32 | 111 | 0 | 1 | 0.9950 | 0.9852 | 1.0000 |
| factor(X25.CS)=0 | 6.33 | 110 | 0 | 1 | 0.9950 | 0.9852 | 1.0000 |
| factor(X25.CS)=0 | 6.35 | 109 | 0 | 1 | 0.9950 | 0.9852 | 1.0000 |
| factor(X25.CS)=0 | 6.36 | 108 | 0 | 1 | 0.9950 | 0.9852 | 1.0000 |
| factor(X25.CS)=0 | 6.37 | 107 | 0 | 1 | 0.9950 | 0.9852 | 1.0000 |
| factor(X25.CS)=0 | 6.38 | 106 | 0 | 3 | 0.9950 | 0.9852 | 1.0000 |
| factor(X25.CS)=0 | 6.39 | 103 | 0 | 3 | 0.9950 | 0.9852 | 1.0000 |
| factor(X25.CS)=0 | 6.42 | 100 | 0 | 3 | 0.9950 | 0.9852 | 1.0000 |
| factor(X25.CS)=0 | 6.46 | 97 | 0 | 1 | 0.9950 | 0.9852 | 1.0000 |
| factor(X25.CS)=0 | 6.47 | 96 | 0 | 4 | 0.9950 | 0.9852 | 1.0000 |
| factor(X25.CS)=0 | 6.48 | 92 | 0 | 1 | 0.9950 | 0.9852 | 1.0000 |
| factor(X25.CS)=0 | 6.54 | 91 | 0 | 1 | 0.9950 | 0.9852 | 1.0000 |
| factor(X25.CS)=0 | 6.55 | 90 | 0 | 1 | 0.9950 | 0.9852 | 1.0000 |
| factor(X25.CS)=0 | 6.56 | 89 | 0 | 1 | 0.9950 | 0.9852 | 1.0000 |
| factor(X25.CS)=0 | 6.57 | 88 | 0 | 1 | 0.9950 | 0.9852 | 1.0000 |
| factor(X25.CS)=0 | 6.6 | 87 | 0 | 1 | 0.9950 | 0.9852 | 1.0000 |
| factor(X25.CS)=0 | 6.61 | 86 | 0 | 1 | 0.9950 | 0.9852 | 1.0000 |
| factor(X25.CS)=0 | 6.64 | 85 | 0 | 1 | 0.9950 | 0.9852 | 1.0000 |
| factor(X25.CS)=0 | 6.65 | 84 | 0 | 1 | 0.9950 | 0.9852 | 1.0000 |
| factor(X25.CS)=0 | 6.68 | 83 | 0 | 1 | 0.9950 | 0.9852 | 1.0000 |
| factor(X25.CS)=0 | 6.69 | 82 | 0 | 1 | 0.9950 | 0.9852 | 1.0000 |
| factor(X25.CS)=0 | 6.71 | 81 | 0 | 1 | 0.9950 | 0.9852 | 1.0000 |
| factor(X25.CS)=0 | 6.73 | 80 | 0 | 1 | 0.9950 | 0.9852 | 1.0000 |
| factor(X25.CS)=0 | 6.75 | 79 | 0 | 2 | 0.9950 | 0.9852 | 1.0000 |
| factor(X25.CS)=0 | 6.77 | 77 | 0 | 1 | 0.9950 | 0.9852 | 1.0000 |
| factor(X25.CS)=0 | 6.78 | 76 | 0 | 2 | 0.9950 | 0.9852 | 1.0000 |
| factor(X25.CS)=0 | 6.81 | 74 | 0 | 1 | 0.9950 | 0.9852 | 1.0000 |
| factor(X25.CS)=0 | 6.83 | 73 | 0 | 1 | 0.9950 | 0.9852 | 1.0000 |
| factor(X25.CS)=0 | 6.85 | 72 | 0 | 1 | 0.9950 | 0.9852 | 1.0000 |
| factor(X25.CS)=0 | 6.86 | 71 | 0 | 2 | 0.9950 | 0.9852 | 1.0000 |
| factor(X25.CS)=0 | 6.87 | 69 | 0 | 1 | 0.9950 | 0.9852 | 1.0000 |
| factor(X25.CS)=0 | 6.89 | 68 | 0 | 1 | 0.9950 | 0.9852 | 1.0000 |
| factor(X25.CS)=0 | 6.91 | 67 | 0 | 1 | 0.9950 | 0.9852 | 1.0000 |
| factor(X25.CS)=0 | 6.95 | 66 | 0 | 1 | 0.9950 | 0.9852 | 1.0000 |
| factor(X25.CS)=0 | 6.96 | 65 | 0 | 3 | 0.9950 | 0.9852 | 1.0000 |
| factor(X25.CS)=0 | 6.98 | 62 | 0 | 1 | 0.9950 | 0.9852 | 1.0000 |
| factor(X25.CS)=0 | 7.14 | 61 | 0 | 1 | 0.9950 | 0.9852 | 1.0000 |
| factor(X25.CS)=0 | 7.16 | 60 | 0 | 1 | 0.9950 | 0.9852 | 1.0000 |
| factor(X25.CS)=0 | 7.2 | 59 | 0 | 1 | 0.9950 | 0.9852 | 1.0000 |
| factor(X25.CS)=0 | 7.26 | 58 | 0 | 2 | 0.9950 | 0.9852 | 1.0000 |
| factor(X25.CS)=0 | 7.29 | 56 | 0 | 1 | 0.9950 | 0.9852 | 1.0000 |
| factor(X25.CS)=0 | 7.35 | 55 | 0 | 3 | 0.9950 | 0.9852 | 1.0000 |
| factor(X25.CS)=0 | 7.36 | 52 | 0 | 4 | 0.9950 | 0.9852 | 1.0000 |
| factor(X25.CS)=0 | 7.37 | 48 | 0 | 1 | 0.9950 | 0.9852 | 1.0000 |
| factor(X25.CS)=0 | 7.38 | 47 | 0 | 1 | 0.9950 | 0.9852 | 1.0000 |
| factor(X25.CS)=0 | 7.39 | 46 | 0 | 2 | 0.9950 | 0.9852 | 1.0000 |
| factor(X25.CS)=0 | 7.4 | 44 | 0 | 1 | 0.9950 | 0.9852 | 1.0000 |
| factor(X25.CS)=0 | 7.42 | 43 | 0 | 1 | 0.9950 | 0.9852 | 1.0000 |
| factor(X25.CS)=0 | 7.47 | 42 | 0 | 1 | 0.9950 | 0.9852 | 1.0000 |
| factor(X25.CS)=0 | 7.48 | 41 | 0 | 2 | 0.9950 | 0.9852 | 1.0000 |
| factor(X25.CS)=0 | 7.5 | 39 | 0 | 1 | 0.9950 | 0.9852 | 1.0000 |
| factor(X25.CS)=0 | 7.58 | 38 | 0 | 3 | 0.9950 | 0.9852 | 1.0000 |
| factor(X25.CS)=0 | 7.59 | 35 | 0 | 1 | 0.9950 | 0.9852 | 1.0000 |
| factor(X25.CS)=0 | 7.6 | 34 | 0 | 2 | 0.9950 | 0.9852 | 1.0000 |
| factor(X25.CS)=0 | 7.61 | 32 | 0 | 1 | 0.9950 | 0.9852 | 1.0000 |
| factor(X25.CS)=0 | 7.64 | 31 | 0 | 1 | 0.9950 | 0.9852 | 1.0000 |
| factor(X25.CS)=0 | 7.67 | 30 | 0 | 1 | 0.9950 | 0.9852 | 1.0000 |
| factor(X25.CS)=0 | 7.69 | 29 | 0 | 2 | 0.9950 | 0.9852 | 1.0000 |
| factor(X25.CS)=0 | 7.73 | 27 | 0 | 2 | 0.9950 | 0.9852 | 1.0000 |
| factor(X25.CS)=0 | 7.81 | 25 | 0 | 1 | 0.9950 | 0.9852 | 1.0000 |
| factor(X25.CS)=0 | 7.82 | 24 | 0 | 1 | 0.9950 | 0.9852 | 1.0000 |
| factor(X25.CS)=0 | 7.9 | 23 | 0 | 1 | 0.9950 | 0.9852 | 1.0000 |
| factor(X25.CS)=0 | 7.94 | 22 | 0 | 1 | 0.9950 | 0.9852 | 1.0000 |
| factor(X25.CS)=0 | 7.99 | 21 | 0 | 1 | 0.9950 | 0.9852 | 1.0000 |
| factor(X25.CS)=0 | 8.06 | 20 | 0 | 1 | 0.9950 | 0.9852 | 1.0000 |
| factor(X25.CS)=0 | 8.07 | 19 | 0 | 1 | 0.9950 | 0.9852 | 1.0000 |
| factor(X25.CS)=0 | 8.09 | 18 | 0 | 1 | 0.9950 | 0.9852 | 1.0000 |
| factor(X25.CS)=0 | 8.26 | 17 | 0 | 1 | 0.9950 | 0.9852 | 1.0000 |
| factor(X25.CS)=0 | 8.29 | 16 | 0 | 1 | 0.9950 | 0.9852 | 1.0000 |
| factor(X25.CS)=0 | 8.37 | 15 | 0 | 1 | 0.9950 | 0.9852 | 1.0000 |
| factor(X25.CS)=0 | 8.41 | 14 | 1 | 0 | 0.9239 | 0.7987 | 1.0000 |
| factor(X25.CS)=0 | 8.5 | 13 | 0 | 1 | 0.9239 | 0.7987 | 1.0000 |
| factor(X25.CS)=0 | 8.53 | 12 | 0 | 1 | 0.9239 | 0.7987 | 1.0000 |
| factor(X25.CS)=0 | 8.58 | 11 | 0 | 1 | 0.9239 | 0.7987 | 1.0000 |
| factor(X25.CS)=0 | 8.87 | 10 | 0 | 1 | 0.9239 | 0.7987 | 1.0000 |
| factor(X25.CS)=0 | 8.88 | 9 | 0 | 3 | 0.9239 | 0.7987 | 1.0000 |
| factor(X25.CS)=0 | 9.02 | 6 | 0 | 1 | 0.9239 | 0.7987 | 1.0000 |
| factor(X25.CS)=0 | 9.04 | 5 | 0 | 1 | 0.9239 | 0.7987 | 1.0000 |
| factor(X25.CS)=0 | 9.06 | 4 | 0 | 2 | 0.9239 | 0.7987 | 1.0000 |
| factor(X25.CS)=0 | 9.08 | 2 | 0 | 1 | 0.9239 | 0.7987 | 1.0000 |
| factor(X25.CS)=0 | 9.61 | 1 | 0 | 1 | 0.9239 | 0.7987 | 1.0000 |
| factor(X25.CS)=1 | 5.4 | 10 | 0 | 1 | 1.0000 | 1.0000 | 1.0000 |
| factor(X25.CS)=1 | 5.81 | 9 | 0 | 1 | 1.0000 | 1.0000 | 1.0000 |
| factor(X25.CS)=1 | 6.2 | 8 | 0 | 1 | 1.0000 | 1.0000 | 1.0000 |
| factor(X25.CS)=1 | 6.51 | 7 | 0 | 1 | 1.0000 | 1.0000 | 1.0000 |
| factor(X25.CS)=1 | 7.27 | 6 | 0 | 1 | 1.0000 | 1.0000 | 1.0000 |
| factor(X25.CS)=1 | 7.72 | 5 | 0 | 1 | 1.0000 | 1.0000 | 1.0000 |
| factor(X25.CS)=1 | 7.74 | 4 | 0 | 2 | 1.0000 | 1.0000 | 1.0000 |
| factor(X25.CS)=1 | 8.63 | 2 | 0 | 1 | 1.0000 | 1.0000 | 1.0000 |
| factor(X25.CS)=1 | 8.69 | 1 | 0 | 1 | 1.0000 | 1.0000 | 1.0000 |

|  |  |  |  |  |  |  |  |  |  |
| --- | --- | --- | --- | --- | --- | --- | --- | --- | --- |
| X25.CS | records | n.max | n.start | events | \*rmean | \*se(rmean) | median | 0.95LCL | 0.95UCL |
| factor(X25.CS)=0 | 241 | 241 | 241 | 2 | 9.079 | 0.054 | NA | NA | NA |
| factor(X25.CS)=1 | 10 | 10 | 10 | 0 | 9.15 | 0 | NA | NA | NA |

Use subset of data: (!is.na(X5) & (X5==1))
Created by EmpowerStats (www.empowerstats.com) and R on 2025-10-07
